# P01.50. Influence of dietary red palm oil on antioxidant status in male Wistar rats

**DOI:** 10.1186/1472-6882-12-S1-P50

**Published:** 2012-06-12

**Authors:** A Ayeleso, O Oguntibeju, N Brooks

**Affiliations:** 1 Cape Peninsula University of Technology, Dept of Biomedical Sciences, Bellville, South Africa; 2 Dept of Wellness Sciences, Cape Peninsula University of Technology, Cape Town, South Africa

## Purpose

The study was carried out to evaluate the antioxidant status in male rats following the dietary consumption of red palm oil.

## Methods

Male wistar rats were randomly divided into three groups. Group 1 (n=5) received no supplementation and served as the control while group 2 (n=6) and 3 (n=6) received 2ml and 4ml red palm oil (RPO), respectively. Plasma total polyphenols, plasma antioxidant capacity [i.e. oxygen radical absorbance capacity (ORAC)], ferric reducing antioxidant power (FRAP) as well as levels of antioxidant enzymes [catalase (CAT), glutathione peroxidase (GPx) and superoxide dismutase (SOD)] were determined using established techniques.

## Results

There were no significant differences (p<0.05) in total polyphenols, ORAC, and FRAP in palm oil fed groups when compared with the control group. Catalase levels significantly increased (p<0.05) at both 2ml and 4ml RPO in the liver and erythrocyte. There was no significant difference in the liver GPx levels in palm oil fed groups while erythrocyte GPx level significantly increased at 4ml RPO when compared with the control group. Red palm oil did not significantly increase (p<0.05) liver and erythrocyte SOD levels in all the groups when compared with the control group.

## Conclusion

Red palm oil did not significantly increase the total antioxidant capacity in the plasma. However, RPO significantly increased the levels of liver and erythrocyte catalase as well as erythrocyte glutathione peroxidase level and hence, its dietary consumption could help to boost antioxidant status in the body and thus promote good health.

